# Proteomic comparison of intact and fetoscopy-induced fetal membrane defect sites

**DOI:** 10.1038/s41390-025-04692-9

**Published:** 2025-12-26

**Authors:** Lukas Moser, Katharina Gegenschatz-Schmid, Eva Avilla-Royo, Ladina Rüegg, Bernd Roschitzki, Jonas Grossmann, Nicole Ochsenbein-Kölble, Martin Ehrbar

**Affiliations:** 1 Department of Obstetrics, University and University Hospital of Zurich, Zurich, Switzerland; 2 University of Zurich, Zurich, Switzerland; 3 Functional Genomics Center, University of Zurich and ETH Zurich, Zurich, Switzerland

## Abstract

**Background:**

Iatrogenic preterm premature rupture of fetal membranes (iPPROM) following fetoscopic interventions remains a major barrier to the advancement of fetal therapies. The mechanisms underlying iPPROM are poorly understood, but the inability of fetal membrane (FM) defects to heal spontaneously likely plays a key role, contrasting with the regenerative potential of amniotic membranes in other contexts.

**Methods:**

To assess the impact of fetoscopic procedures on FMs, tissue samples from patients who underwent laser surgery for twin-to-twin transfusion syndrome (16–27 weeks gestation, *n* = 8) were collected after cesarean delivery at 29–35 weeks. Samples were categorized by proximity to the trocar site and analyzed using proteomic and histological methods.

**Results:**

While differential expression analysis in the amnion revealed no significant changes, pathway enrichment indicated increased collagen deposition at defect sites. In the chorion, seven differentially expressed proteins were identified, largely linked to enhanced intercellular contact stability. These findings suggest the amnion may respond to mechanical stress by reinforcing structural integrity through collagen deposition, while the chorion may attempt to stabilize cell junctions. However, no other signs of tissue regeneration were observed.

**Conclusion:**

This study provides molecular and cellular evidence that FMs lack a substantial healing response post-surgery, underscoring the need for biologically informed repair strategies.

**Impact:**

- By combining untargeted proteomics with histological and qPCR evaluations, this study demonstrates subtle molecular and cellular changes in fetoscopy-induced fetal membrane defects at the time of delivery.
- This indicates minimal molecular and cellular healing mechanisms in the fetal membranes.
- This underscores the potential for sealing FM defects after fetoscopy to prevent amniotic fluid leakage, thereby reducing the incidence of intra-amniotic preterm rupture of membranes.

## Introduction

Fetoscopic interventions have emerged as a vital tool in modern prenatal medicine, enabling the diagnosis and treatment of various fetal conditions in utero. These procedures are particularly crucial for managing complications such as twin-twin transfusion syndrome (TTTS), congenital diaphragmatic hernia, and spina bifida.^1^ In the case of TTTS, naturally conceived monozygotic twins occur in approximately 1 in 250 to 1 in 400 pregnancies, and among these, 10–15% develop the syndrome. Data from Germany indicate that, specifically for TTTS, an average of around 159 fetal laser therapies were performed annually between 2005 and 2021, with numbers increasing over time.^2^ However, these procedures are not without risks. A major concern is the occurrence of iatrogenic preterm prelabor rupture of the fetal membranes (iPPROM), which frequently leads to preterm birth and its associated neonatal morbidity and mortality.^3^ iPPROM is reported to occur in approximately 30% of cases, although some studies have documented rates as high as 60–100%.^4^ Unfortunately, despite its high incidence, no clinical interventions currently exist to prevent this complication.^5^

The fetal membranes (FMs), composed of the amnion and the chorion, are essential for maintaining fetal health throughout pregnancy. The amnion consists of epithelial cells that are in direct contact with the amniotic fluid and rest on a basement membrane. Beneath this lies a thicker fibroblast layer that serves as the primary load-bearing structure and contains amniotic mesenchymal stromal cells. The spongy layer of the amnion interfaces with the chorion, the thicker of the two layers. The chorion is composed of three sublayers: the reticular layer, which contains numerous Hofbauer cells and some fibroblasts; a basement membrane layer; and a trophoblast layer that is responsible for maintaining immunological tolerance.^4^

The growth and the stability of the FMs until parturition are maintained by a delicate balance between extracellular matrix (ECM) deposition and degradation, mediated by an interplay between matrix metalloproteinases (MMPs) and their inhibitors.^6^ The current hypothesis is that chorion-amnion separation leads to a weakening of the FMs and may result in their rupture.^7^ Studies suggest that reduced lubrication between the amnion and chorion increases friction, leading to higher mechanical stresses in the amnion and potentially contributing to PPROM.^8^ Additionally, there is growing evidence that aging of the FMs, along with ECM remodeling and degradation, contributes to FM rupture prior to parturition.^9^

Despite significant research, the precise mechanisms underlying iPPROM remain unclear. Several theories have been proposed, with the most common including postoperative chorion-amnion separation, membrane apoptosis, or damage at the trocar (defect) site.^10^ It is known that fetoscopically-induced FM defects do not heal and can still be observed at birth.^11^ This stands in stark contrast to the successful use of FMs as healing-supportive scaffold, including burn wound healing, treatment of hard-to-heal wounds such as foot ulcers, and even in research on neuronal damage.^12–14^ Importantly, altered collagen morphology and increased apoptosis have been found in FM defects.^15^ However, a comprehensive analysis of the molecular composition at the defect site is still lacking. Given that the ECM in the FMs undergoes constant remodeling, it is critical to understand how this process is affected at the defect site. Additionally, it remains unknown whether the FMs at the defect site experiences heightened cellular senescence and sterile inflammation, which are implicated in spontaneous PPROM.^16,17^

This study aims to fill this gap by employing liquid chromatography-mass spectrometry (LC-MS) proteomics. LC-MS proteomics is a technique that separates proteins-derived peptides by liquid chromatography and identifies them using mass spectrometry. This allows for an unbiased, high-throughput analysis of protein expression in complex tissue samples. We compared the defect sites to control regions and identified changes in protein expression occurring at the defect site in an unbiased manner. While the number of differentially expressed proteins was limited, the study provides a foundational dataset for future investigations. It also highlights the technical and biological challenges in detecting subtle local changes following fetoscopic procedures. These insights contribute to a more nuanced understanding of the tissue environment at the defect site and may inform future strategies to assess or mitigate iPPROM risk.

## Results

### Study design and sample collection

**Fig. 1:**
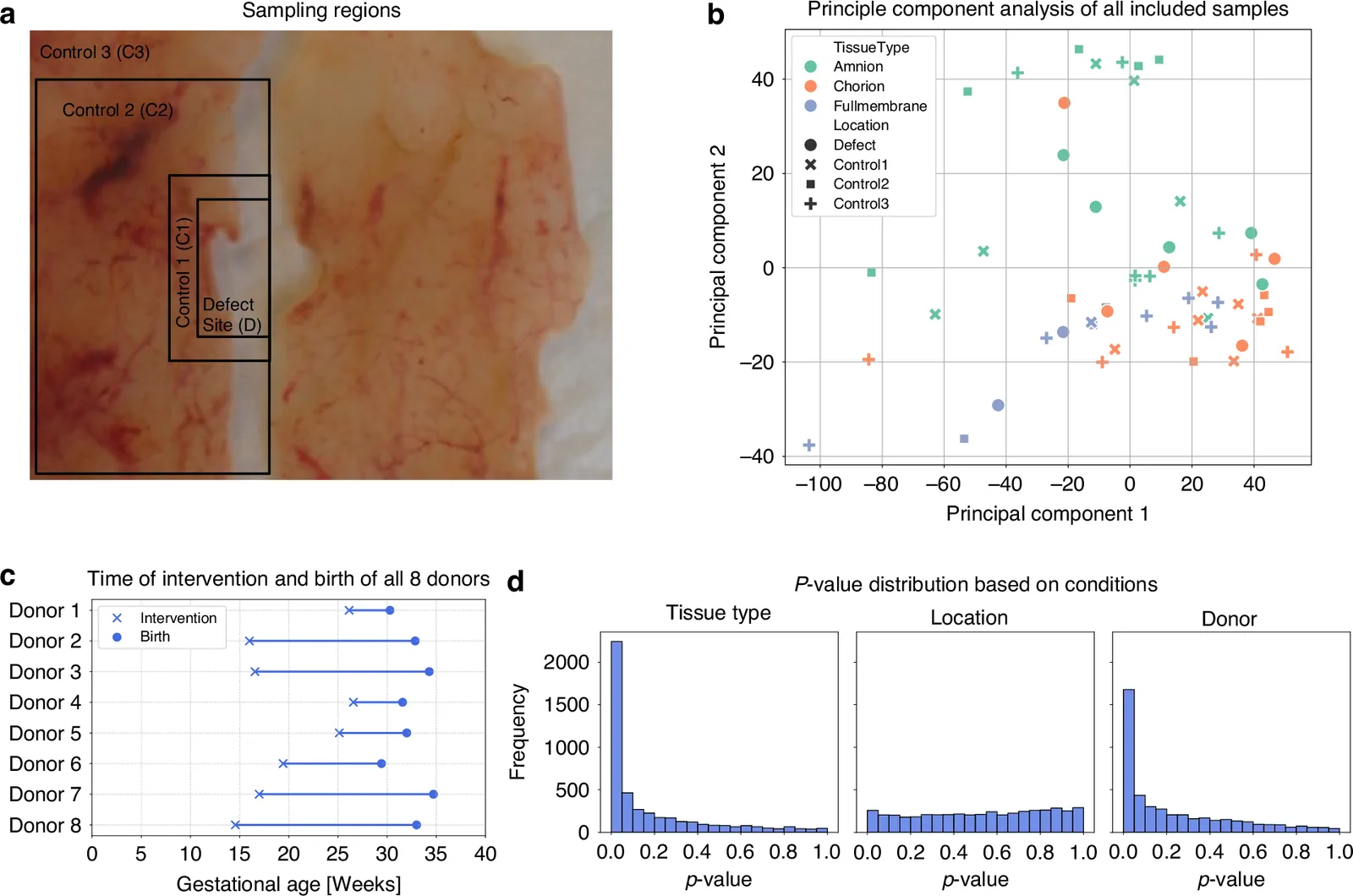
Study design and overview of FM sampling and proteomic profiling. **a** Overview of a FM after delivery, highlighting the fetoscopically induced defect site. The blue lines indicate the regions of interest, which were sectioned for analysis. One side of each section was used for proteomics, and the other for histology. **b** Principal Component Analysis (PCA) plot of normalized proteomics data, colored by tissue type (chorion, amnion, and full membrane) and shaped according to the position within the FM. **c** Gestational age at fetoscopic intervention (x) and delivery (o) for each donor. **d** Histogram of the distribution of *p*-values for differentially expressed proteins, grouped by tissue type (chorion, amnion, full membrane), location (defect site, control 1, control 2, control 3), and donor.

Eight patients (in the following called donors) undergoing fetoscopic laser surgery for twin-to-twin transfusion syndrome (TTTS) were included in this study (details in Supplemental Table S1). To capture proteomic changes induced by the intervention, membrane samples were collected at varying distances from the surgical site (see Fig. 1a). Samples were obtained from surgeries conducted between 16 and 27 weeks of gestation. Correspondingly, gestational age at delivery ranged from 29 to 35 weeks, with intervals between surgery and delivery spanning less than 5 to over 17 weeks (Fig. 1c & Supplemental Table S4).

### Proteomic profiling and data processing

Label-free liquid chromatography coupled to mass spectrometry (LC-MS) was employed to characterize protein expression. Data were analyzed and normalized using DIA-NN^18^ and Prolfqua,^19^ identifying a total of 4727 proteins. Principal component analysis (PCA) showed clear separation between amnion and chorion, with intact FM samples (where amnion and chorion were not separated) clustering between them. However, PCA did not reveal clustering by proximity to the defect site (Fig. 1b).

Differential expression analysis highlighted tissue type as the primary driver of proteomic variance, indicated by a distinct peak in the *p*-value distribution below 0.05. Donor-specific variability was also present, albeit to a lesser extent. In contrast, p-values associated with location showed a uniform distribution, suggesting minimal proteomic differences near the defect site versus distant regions (Fig. 1d).

Given the tissue heterogeneity, further analyses were conducted separately for each tissue type. Additionally, a mixed model was implemented to reduce donor-related variability by performing paired analyses and considering the donor as a random variable.

### Amnion: collagen formation and structural changes

**Fig. 2:**
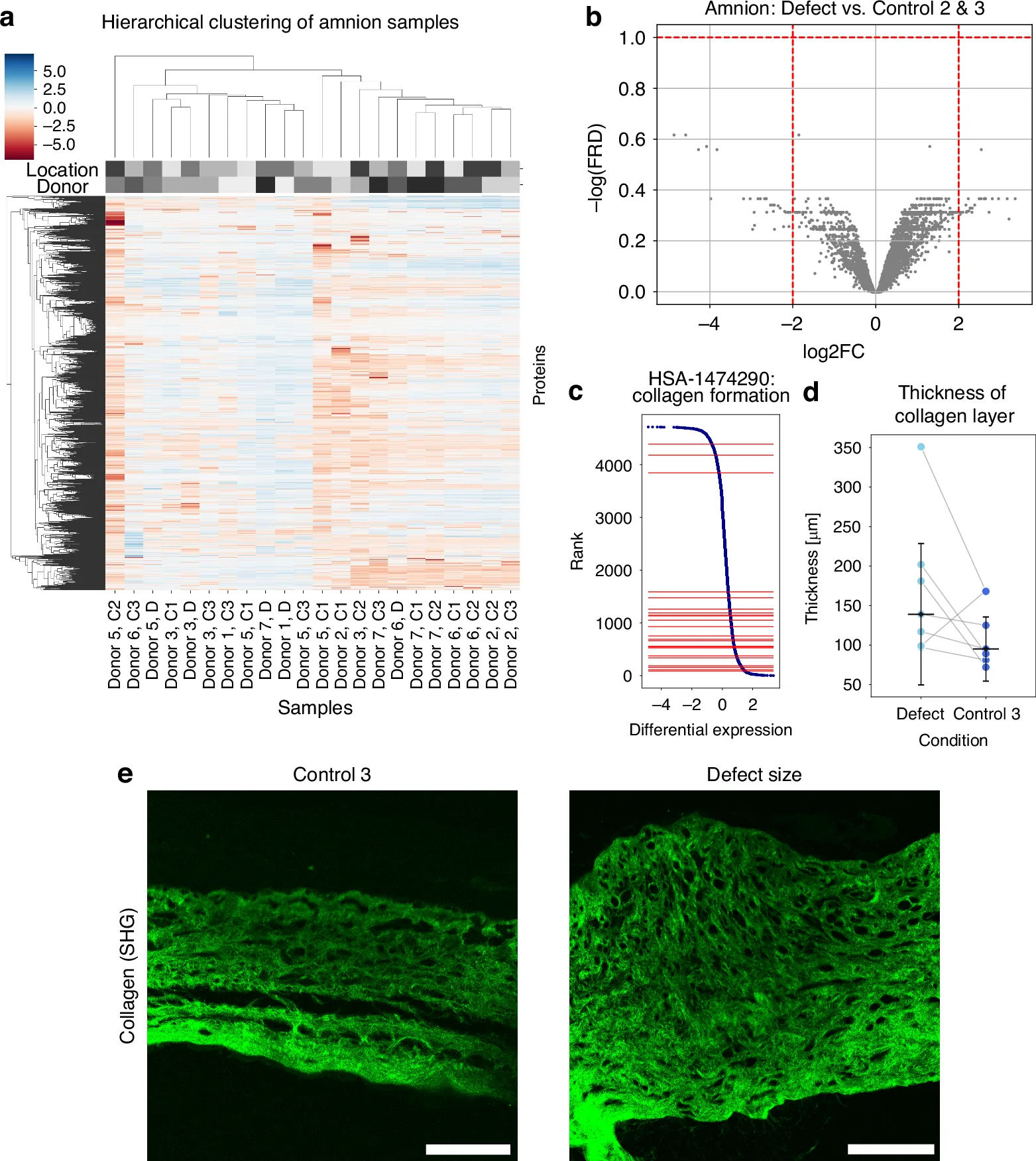
Differential protein expression and collagen remodeling in the amnion at the fetoscopic defect site. **a** Heatmap of normalized protein abundance, focusing exclusively on amnion samples. Clustering of proteins and donors was performed using Euclidean distance and Ward’s method (H = Defect Site, C1 = Control 1, C2 = Control 2, C3 = Control 3). The same grey color indicate either the same donor or the same location. The corresponding donor or location can be found in the x-axis label. **b** Volcano plot comparing differentially expressed proteins between the fetoscopically induced defect site and control regions 2 and 3 in the amnion layer. (Red line indicates a log fold change of 2 and a –log(FDR) of 1). **c** This plot shows the ranked distribution of proteins based on differential expression. The x-axis represents the log₂ fold change of proteins, and the y-axis shows the rank accordingly. Proteins associated with significantly enriched REACTOME term “Collagen formation” identified by STRING are highlighted with a horizontal red line. Higher ranks indicating higher expression at the defect site. **d** Measurement of collagen layer thickness in histology slides, using second harmonic generation imaging, comparing the defect site and control region 3. The black bar indicate the mean and the standard deviation. The paired samples are connected with a grey line. **e** Representative images of the collagen layer obtained via second harmonic generation imaging. The left image shows control region 3, and the right image shows the sample from the defect site. (scale bar = 100 μm)

In the amnion, heatmap analysis did not reveal clustering based on either donor or location. Proximity to the defect site did not result in clustering, as shown by the heatmap’s color distribution (Fig. 2a). A volcano plot comparing the defect site with distant controls (controls 2 and 3, excluding control 1 for clarity) did not reveal any significantly upregulated or downregulated proteins near the defect site when applying a 10% false discovery rate (FDR) (Fig. 2b).

However, functional enrichment analysis via STRING’s20 rank based tool identified “Collagen Formation” as a significantly enriched REACTOME^20^ pathway (*p* ≤ 0.05). A rank-based plot showed several collagen-associated proteins were upregulated near the defect site (Fig. 2c). Second harmonic generation imaging supported this, indicating increased collagen thickness in these regions (Fig. 2d). While paired comparisons in most cases (5/6) showed higher collagen content near the defect site compared to the control site 3, this trend did not reach statistical significance (Fig. 2e).

### Chorion: cornified envelope development

**Fig. 3:**
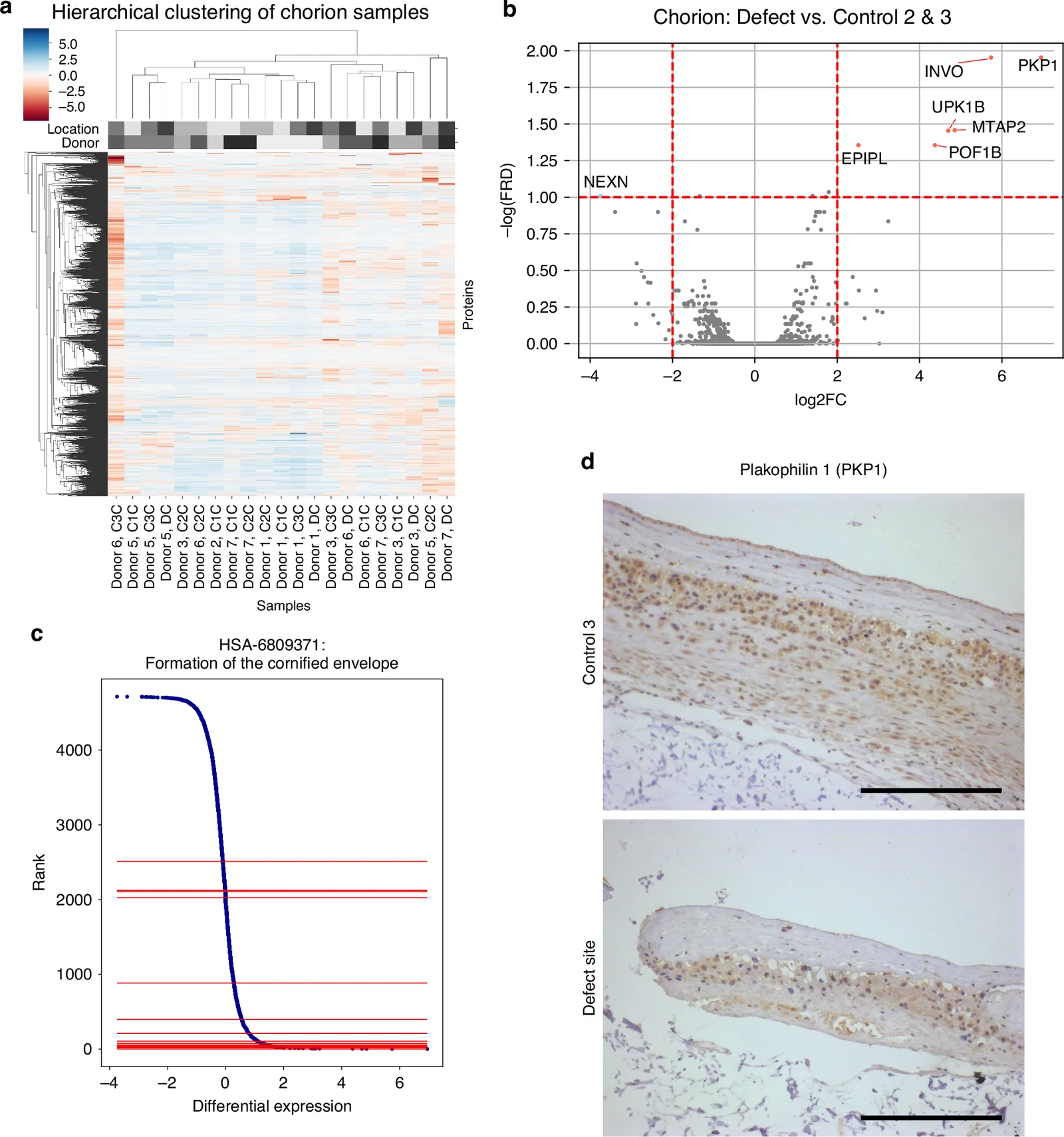
Proteomic and histological characterization of chorion response at the defect site. **a** Heatmap of normalized protein abundance, focusing exclusively on chorion samples. Clustering of proteins and donors was performed using Euclidean distance and Ward’s method (H = Defect Site, C1 = Control 1, C2 = Control 2, C3 = Control 3). The same grey color indicate either the same donor or the same location. The corresponding donor or location can be found in the x-axis label. **b** Volcano plot showing differentially expressed proteins between the fetoscopically induced defect site and control regions 2 and 3 in the chorion layer. (Red line indicates a log fold change of 2 and a –log(FDR) of 1). **c** This plot shows the ranked distribution of proteins based on differential expression. The x-axis represents the log₂ fold change of proteins, and the y-axis shows the rank accordingly. Proteins associated with significantly enriched REACTOME term “Formation of the cornified envelope” identified by STRING are highlighted with a horizontal red line. Higher ranks indicating higher expression at the defect site. **d** Expression of Plakophilin 1 (PKP1) (brown) in histological samples from the control 3 (top) and the defect site (bottom) regions. (scale bar = 200 μm)

As with the amnion, heatmap analysis in chorion samples did not demonstrate clustering by donor or location (Fig. 3a). However, a volcano plot comparing the defect site to distant controls revealed several significantly upregulated proteins, namely: Plakophilin-1 (PKP1) a protein known to recruit numerous desmosomal components,^21^ Involucrin (INVO) a precursor of the cornified envelope, which is stable and insoluble structure forming below the plasma membrane of terminally differentiated cells,^22^ Uroplakin 1B (UPK1B) a molecule known to improve the mechanical stability of the bladder,^23^ Microtubule Associated Protein 2 (MTAP2), Premature Ovarian Failure Protein 1B (POF1B) a protein co-localizing with tight junctions,^24^ and Epiplakin 1 (EPIPL) maintaining intermediate filaments under stress.^25^ The only significantly downregulated protein was Nexilin (NEXN) a protein involved in smooth muscle cell adhesion, and migration^26^ (Fig. 3b). While of these, PKP1 and EPIPL consistently showed upregulation across donors at the defect site, the other proteins were only differentially expressed in 1 out of 5 donors (Supplemental Fig. S2). STRING’s^27^ rank based tool for functional enrichment analysis identified “Cornified Envelope Development” as a significantly enriched REACTOME^20^ as shown in a rank-based plot (Fig. 3c). Associated proteins were involved in cell-cell interactions, particularly desmosomes and hemi desmosomes. Despite these findings, immunostaining for PKP1, showed no clear difference between intervention and control regions.

### Structural and cellular observations

**Fig. 4:**
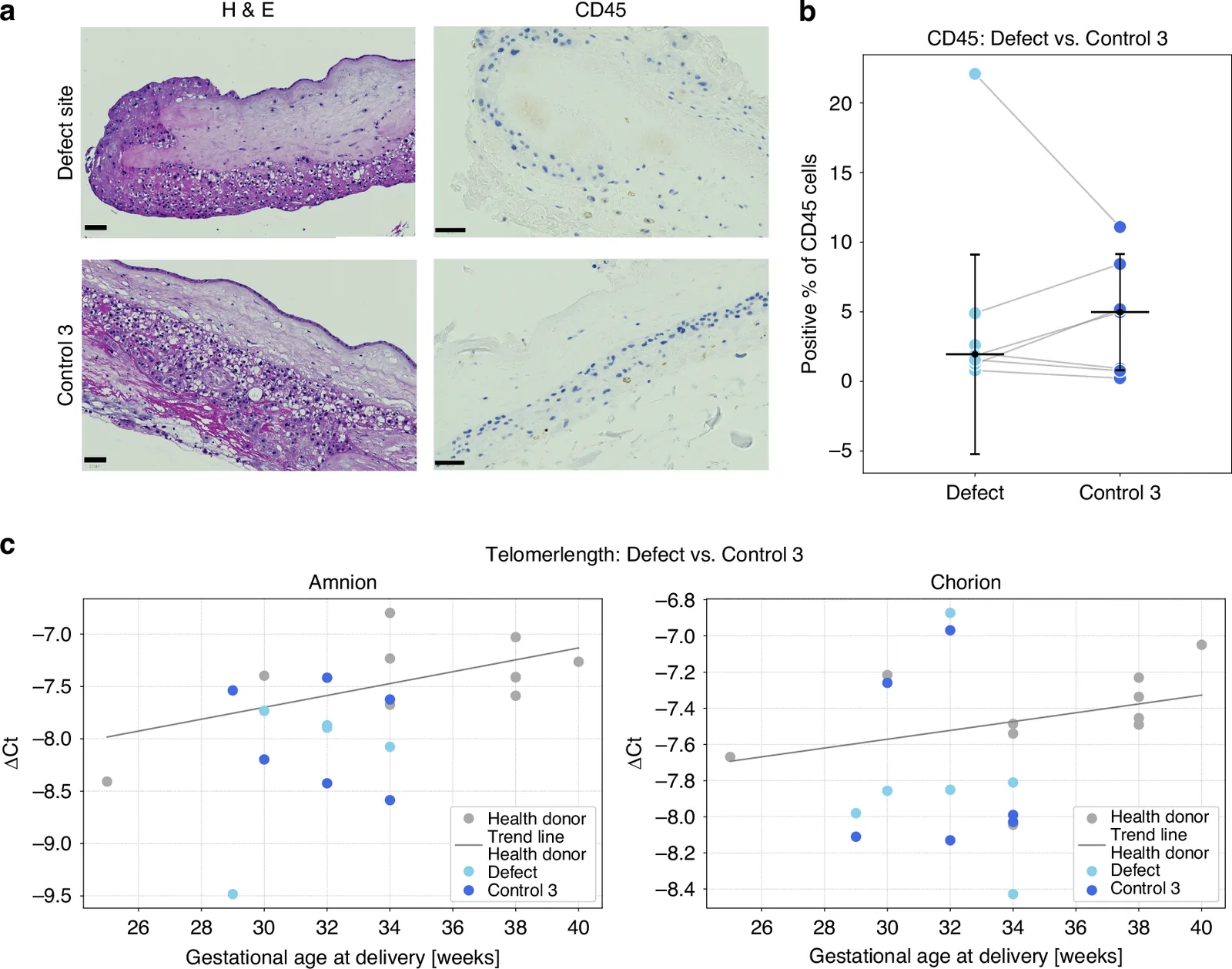
Assessment of immune cell infiltration and telomere length in FMs at the defect site. **a** Hematoxylin and Eosin staining of the full FM at the fetoscopically induced defect site (top) and control region 3 (bottom). CD45 staining (brown) of the full FM, comparing the defect site (top) and control region 3 (bottom). (scale bar = 50 μm). **b** Quantification of CD45-positive cells as a percentage of all cells at the hole and control region 3. The black bar indicates the mean and the standard deviation. The grey line combines the paired samples. **c** Telomere length measured by PCR in amnion and chorion samples from the hole and control region 3 as a delta fold change normalized to 36B4, with additional comparisons to healthy control samples (shown in grey and its trend line).

Hematoxylin and eosin staining revealed structural differences between regions, particularly in the chorion, where in some cases a wrapping around the amnion was observed (Supplementary Fig. S3). However, CD45 immunostaining for immune cells showed no significant differences between defect sites and control regions (Fig. 4a). Quantification of CD45-positive cells indicated a slight, non-significant increase in immune presence in control 3 samples, but paired comparisons showed no consistent pattern (Fig. 4b).

Quantitative PCR analysis of telomere length in healthy donor samples confirmed a trend of shortening with advancing gestational age in both amnion and chorion. However, comparisons between samples from the defect site and distant controls (control 3) revealed no consistent difference in telomere length. Both groups exhibited telomere lengths similar to those of healthy donor tissues (Fig. 4c).

## Discussion

We investigated the potential healing-modulating responses of fetoscopically injured FMs by combining a non-targeted proteomic approach with histological observations. The absence of distinct clustering between control and puncture sites suggests the lack of a broad healing response. However, the observed increase in collagen thickness near the amnion puncture site, along with the presence of cornified envelope-related proteins PKP1 and EPIPL in the chorion, points to a mechanical reinforcement of the FM defect area. Moreover, the lack of immune cell recruitment and telomere shortening at the injury site indicates the absence of immune activation or accelerated premature cellular senescence.

Fetoscopy-induced FM defects, when analyzed histologically at the time of delivery, showed a very local morphological change, which, in agreement with previous studies, is typically the coverage of the amnion by chorion tissue (Supplemental Fig. S3).^11,15^ As previously discussed, this chorion coverage could be the result of trophoblasts migrating and proliferation from the adjacent chorion.^15^ It could also be due to the direction of puncture, which may cause the chorion to slide over the amnion. These histological observations together with the minimal changes of protein expression in FM defect sites compared to control sites, here shown by global clustering, heatmap, and PCA analyses, suggest the occurrence of very restricted and minimal regenerative responses.

Evaluations focusing on amnion protein abundances substantiated the absence of significant changes as only rank-based evaluations revealed a functional enrichment related to collagen formation. This, as well as the increased thickness of the collagen layer are consistent with prior findings showing a long-term structural adaptation of collagen at the defect site.^15^ In contrast, previous studies reported increased expression of connexin 43 (Cx43) at FM defect sites. This discrepancy may be explained by the higher spatial resolution of their imaging and PCR-based methods, which allow for more precise sampling of the defect area and may detect subtle, localized changes.^5^ Notably, Papanne et al. also did not observe major alterations at the defect site.^15^ Taken together, these findings support the hypothesis that in the amnion, any healing-related processes are minimal and insufficient to trigger a typical regenerative response. However, it would be valuable to elucidate what triggers the observed collagen reinforcement, particularly which molecular pathways or mechanosensory processes initiate this response. This could elucidate whether such localized structural adaptation is linked to or even contributes to the impaired healing of fetal membranes and how it could be overcome.

In the chorion significantly upregulated proteins in the FM defect sites were related to cell adhesion, cytoskeleton, and tissue integrity. Additionally, significant changes were related the REACTOME term “Development of the Cornified Envelope” comprising proteins involved in the formation of a rigid and insoluble protein scaffold that stabilize terminally differentiated keratinocytes.^28^ The most differentially upregulated protein PKP1, through its role in desmosomes junction formation, can support the maintenance of tissue integrity under mechanical stress. The upregulation of desmosomes could be due to the puncture-induced tissue tension^29^ and may reinforce the membrane defect.^30^ EPIPL, the other reproducibly upregulated protein, by supporting intermediate filament stability, could support the maintenance of tissue tension. However, both PKP1 and EPIPL were described to inhibit cell migration and could restrict FM healing by blocking the mobilization of chorionic cells into the defect.^31–33^ To better understand the connection between impaired healing, the upregulation of the cornified envelope, and broader cellular responses, future studies employing spatial transcriptomics or single-cell proteomics could provide valuable insight into local cellular heterogeneity and signaling within the defect region. Overall, these findings suggest that the chorion may compensate the change in local mechanics by stabilizing cell-cell interaction, while potentially hindering cell migration and contributing to the lack of healing. However, as amnion and chorion layers might be tightly attached to each other, the identified proteins may also result from contamination with amnion cells. Additionally, due to high background signals of immunohistochemically stained chorion the differential expression of proteins could not be confirmed histologically, such that false positive findings cannot be completely excluded.

From a mechanical standpoint, the observed increase in collagen deposition and the upregulation of cornified envelope–related proteins such as PKP1 and EPIPL may represent two non–mutually exclusive scenarios: a local adaptive reinforcement intended to stabilize the defect by reducing strain, or a maladaptive stiffening that increases local stress concentrations at the interface between the reinforced and compliant regions. Our proteomic and histologic data alone cannot discriminate between these hypotheses, highlighting the need for targeted biomechanical analyses such as atomic force microscopy to map local stiffness or ex vivo tensile testing to evaluate rupture mechanics.

It is currently hypothesized that telomere shortening acts as a molecular clock, with senescence and inflammation taking over at a certain point, leading to birth. Stress can accelerate telomere shortening, inducing preterm birth.^6^ We did not observe a clear shortening of telomere length between the defect sites and control 3 sites. Moreover, we did not observe a general telomere shortening after TTTS, which may indicate that this mechanism is not the primary driver of iPPROM. However, consistent with previous findings,^15^ we did neither find signs of inflammation nor an increased immune cell recruitment in the evaluated FM defects.

The major limitations of this study are the heterogeneity in gestational age at surgery and at delivery as well as low number of included patients. This restricts the statistical power to detect differences between the defect site and control sites, particularly given the high donor-to-donor variability. Increasing the number of donors would enhance the statistical power. While a larger cohort could uncover more subtle distinctions, we believe that any major differences would have been apparent in our analysis. Therefore, the lack of observed significant differences suggests that any existing variations are likely minor and may still offer valuable insights into the non-healing nature of the FM. Differences based on gestational age and time of delivery are challenging for the detection of significant changes, however, they enhance the generalizability of our findings.

Despite these limitations, our study suggests that no pronounced adverse reactions, such as strong inflammatory or healing responses at the defect site would directly lead to iPPROM. Instead, more likely other factors, such as the leakage of amniotic fluid, may play a significant role in the development of iPPROM. In this context, it would be important to investigate whether exposure of the decidua to amniotic fluid triggers cellular or inflammatory reactions that could trigger the weakening of the fetal membranes. Understanding how decidual cells respond to amniotic fluid leakage, whether through altered extracellular matrix remodelling, inflammation, or signalling to the chorion, could reveal indirect pathways contributing to FM rupture. This supports the notion that conventional closure techniques, such as suturing the wound, may be sufficient to mitigate the risk of iPPROM. This aligns with ongoing clinical trials investigating the efficacy of surgical closure strategies.^34^

This study provides novel insights into the molecular response of FMs at the defect site following fetoscopic interventions. Despite a small sample size and inherent donor variability, the findings suggest that the incision site exhibits minimal adverse reactions, characterized by the absence of significant inflammation, cellular senescence, or widespread extracellular matrix remodeling at the defect site. These results indicate that the FM’s response to puncture is limited, with only subtle molecular changes observed, such as localized collagen accumulation in the amnion and upregulation of structural proteins related to cornified envelope formation in the chorion. Importantly, the lack of a detectable healing response until delivery highlights two key considerations. First, the tissue at the defect site may not be a primary driver of iatrogenic preterm premature rupture of membranes (iPPROM) through mechanical weakening alone, suggesting that other factors, such as amniotic fluid leakage or biomechanical stress, may play a more substantial role. Second, the absence of natural tissue repair underscores the necessity for artificial closure techniques to restore membrane integrity. Taken together, these findings support the development and refinement of targeted surgical strategies to close FM defects and reduce the risk of iPPROM, ultimately improving perinatal outcomes after fetoscopic procedures.

## Methods

### Tissue collection

Tissue samples from twin-to-twin transfusion syndrome (TTTS) cases and control samples for PCR (preterm and term) were collected post-cesarean section with informed written consent, as approved by the Ethical Committee of the District of Zurich (Approval Number: BASEC_Nr: 2023-00110). The trocar entry site was always on the recipient side, and samples were collected from that location. Tissue samples for TTTS (*n* = 8) were collected in phosphate-buffered saline (PBS, Gibco, Thermo Fisher Scientific, Waltham, MA), following the procedure outlined in Fig. 1a. Donor 1 and 4–7, were diagnosed with iPPROM prior to cesarean section. Donor 1 was monochorionic and monoamniotic, all others were monochorionic and diamniotic. All donors received two doses of 12 mg each of either dexamethasone or betamethasone administered intravenously, 24 h apart; the exact timing of administration relative to birth is detailed in Table SI4. Donor 4 reveived a rescue dosis shortly before birth (same amount as the other dosis). The amnion and chorion layers were carefully separated, snap-frozen in liquid nitrogen, and stored at −80 °C until further use. The details of which donors were included in each experiment are provided in the supplementary information. The preterm and term controls for PCR were also collected after ceasearen section and washed in PBS. Afterwards the tissue was separated in amnion and chorion by blunt dissection and pieces of around 90 mg were snap-frozen and stored at −80 °C until further use. Exclusion criteria included mothers with HIV, hepatitis B, or chromosomal abnormalities. Midterm FMs used as PCR controls (*N* = 1) were obtained during the surgical repair of spina bifida (myelomeningocele, MMC), with written consent and ethical approval from the District of Zurich (KEK Nr. 2015-0247).

### Protein extraction

Tissue samples were ground using a mortar and pestle on dry ice. For each milligram of tissue, 5 µL of lysis buffer (0.1 M Tris-HCl, pH 7.8, 4% sodium dodecyl sulfate (SDS)) was added. 30 mg glass beads (SIGMA G9268-250G, Sigma-Aldrich, St. Louis, MO) were included per 50 µL of lysis buffer. Samples were lysed using a Qiagen Retsch TissueLyser II (two cycles of 2 min at 30 Hz). Following lysis, samples were centrifuged at 14,680 rpm for 10 min, and the supernatant was collected. Protein concentrations were measured using UV-Vis spectrophotometry (280 nm) on a DeNovix DS11 spectrophotometer. Samples were then stored at −20 °C until further analysis.

Upon thawing, samples were sonicated for seven minutes. Protein concentrations were standardized to 25 µg per sample and diluted to a final volume of 50 µL with lysis buffer. If samples exhibited a gel-like consistency, they were incubated for ten minutes at 90 °C to ensure complete solubilization.

### Protein digestion

Protein digestion was carried out using a KingFisher system. Briefly, a bead plate was prepared with a mixture of hydrophobic and hydrophilic beads diluted (GE Life Sciences; GE65152105050250, GE45152105050250) in water to a final concentration of 5 µg/µL. The binding plate contained 50 µL of cell lysate, which was filled up to 100 µL with ethanol (final concentration: 0.25 µg/µL). Wash plates were prepared with 50 µL of 80% ethanol. Elution plate 2 was prepared by adding 100 µL of water to each well, while elution plate 1 contained 25 mM triethylammonium bicarbonate (TEAB) in water with trypsin (1000X dilution, PROMEGA, Promega, Madison, WI), and 150 µL per well was added.

The KingFisher system was set to stop after processing elution plate 1, and the plate was incubated overnight at 37 °C with shaking (300 RPM). After incubation, the plate was centrifuged at 500 g for 30 s before being placed back into the KingFisher for program continuation. Post-program, the eluted liquids from plates 1 and 2 were combined. The enzymatic reaction was stopped by adding trifluoroacetic acid (TFA) to a final concentration of 0.5%. Any precipitate was removed by centrifugation, and the supernatant was filtered using StageTips^35^ to remove residual beads and debris.

StageTips were prepared by first loading 150 µL of 100% acetonitrile (ACN) into each tip, centrifuging at 2000 × g for one minute, and discarding the flow-through. The tips were then equilibrated with 150 µL of 60% ACN and 0.1% TFA, centrifuged at 2000 × g for one minute, with the flow-through discarded. Up to 300 µL of peptide solution was loaded onto the StageTips and centrifuged at 2500 × g for three minutes. Peptides were eluted with 150 µL of 60% ACN and 0.1% TFA, centrifuged at 2500 × g for three minutes. The eluted peptides were dried using a speed-vac set to 36 °C for approximately three hours and stored at −20 °C until further analysis.

### LC-MS/MS Analysis

Peptide samples were reconstituted in 20 µL of MS buffer containing 3% acetonitrile and 0.1% formic acid in water, then diluted to a concentration of 0.1 µg/µL. iRT peptides (Biognosys, Schlieren, Switzerland) were spiked into each sample. A volume of 3 µL of the peptide solution was loaded onto a nanoAcquity UPLC system (Waters, Milford, MA) interfaced with a Orbitrap Fusion Lumos Tribrid Mass Spectrometer. Peptide separation was performed on a C18 column (Acquity UPLC M-Class HSS T3 C18, 1.8 µm, 75 µm x 250 mm, Waters) at 50 °C. The gradient program was as follows: 5%–40% solvent B over 0−90 min, 95% solvent B from 90-95 minutes, and 5% solvent B from 95 to 105 min. The sample injection order was randomized, with a quality control sample injected every four runs.

For the analysis of the individual samples, the mass spectrometer was operated in data-independent mode (DIA). DIA scans covered a range from 396 to 960 m/z in windows of 8 m/z. The resolution of the DIA windows was set to 15’000, with an AGC target value of 500’000, the maximum injection time set to 22 ms and a fixed normalized collision energy (NCE) of 33%. Each instrument cycle was completed by a full MS scan monitoring 396 to 1000 m/z at a resolution of 60’000.

### Protein identification and quantification

Protein quantification was performed using DIA-NN (v1.8.1.8)^18^ with a false discovery rate (FDR) threshold of 1%. Peptides were generated through tryptic digestion, allowing cleavage after Lysine (K) and Arginine (R), with a minimum peptide length of 6 amino acids and a maximum of 30 amino acids. Up to one missed cleavage was permitted. Oxidation of Methionine (UniMod:35) and a mass shift of 15.994915 Da on Methionine were considered as variable modifications, with only one modification per peptide allowed.

Data analysis was conducted using the prolfqua R package (version 1.2.5).^19^ Logarithmic scaling followed by robust scaling (robscale) was used for data transformation. Peptide abundances were aggregated using the median polish method to estimate normalized protein abundance. A linear model was employed to evaluate the effects of tissue type, location, and donor on protein abundance, with a mixed linear model incorporating donor as a random effect (TissueType * Location + Donor). This model was used to identify differentially expressed proteins and their significance. Volcano plots, as all other plots in this manuscript, were generated in Python (3.12.3), and heatmaps were created using Seaborn (0.13.2) with Euclidean distance metric and Ward’s method.^36^ Proteins of interest were identified by applying the log fold change of proteins to the STRING^27^ value/rank based functional enrichment analysis tool, and the significantly expressed REACTOME^20^ terms (*p*-value ≤ 0.05) were displayed in a rank-based plot.

### Histology

For histological analysis, FMs were placed onto a collagen sponge (Lyostypt, Braun, Melsungen, Germany) and fixed in 4% formalin for 2 hours at room temperature. Following fixation, tissues were dehydrated through a graded ethanol series, cleared, and embedded in paraffin (Paraplast, Leica, Muttenz, Switzerland). The paraffin blocks were placed in standard histological cassettes. Sections were cut at a thickness of 4μm and stained with hematoxylin and eosin (H&E) using standard protocols. For immunohistochemical (IHC) staining, antigen retrieval was performed by incubating sections in Target Retrieval Solution, High pH 9 (K8004, DAKO, Glostrup, Denmark) for 20 min at 97 °C using the Dako PT Link system (PT100/PT101, DAKO). IHC staining was carried out using the Dako Autostainer Link 48 (DAKO) according to the manufacturer’s protocol. The following primary antibodies were used: CD45 (Ready-to-Use, IR751, DAKO) and PKP1 (1:100, Abcam ab1835, Cambridge, UK). Detection was performed using the EnVision HRP secondary antibody system (DAKO) for mouse or rabbit antibodies, as appropriate. Hematoxylin was used as a counterstain. Whole-slide images were acquired using a Zeiss Axio Scan Slide Scanner. Quantification of CD45-positive cells was performed using QuPath software.

### PCR analysis

DNA was extracted using the Roche High Pure PCR Template Preparation Kit (Roche, Basel, Switzerland), according to the manufacturer’s protocol. Tissue samples between 30 and 90 mg were used. DNA concentration was measured with a Nanodrop spectrophotometer, and samples were stored at −20 °C. PCR was conducted using the QuantiTect SYBR Green PCR Kit (Cat. No 204143, Qiagen, Hilden, Germany) with primers obtained from Microsynth (Balgach, Switzerland). The following primers were used Telomere 1: GGTTTTTGAGGGTGAGGGTGAGGGTGAGGGTGAGGGT, Telomere 2: TCCCGACTATCCCTATCCCTATCCCTATCCCTATCCCTA, *36B4u*: CAGCAAGTGGGAAGGTGTAATCC, *36B4d*: CCCATTCTATCATCAACGGGTACAA.^37^ The RT-qPCR was done at the ViiA 7 Real-Time PCR System (Applied Biosystems). For the telomere primers, the program began with an initial denaturation step of 15 min at 95 °C, followed by 25 cycles of 15 s at 95 °C, 2 min at 54 °C, and 30 s at 72 °C. For the *36B4* primers, the program also began with an initial 15 min step at 95 °C, followed by 35 cycles of 15 s at 95 °C, 2 min at 58 °C, and 30 s at 72 °C.

## Supplementary information

Supplementary Information

## Data Availability

The mass spectrometry proteomics raw data and DIANN output have been deposited to the ProteomeXchange Consortium via the PRIDE (http://proteomecentral.proteomexchange.org) partner repository with the dataset identifier PXD067401. The scripts for the data analysis in Python and R can be found on Github (https://github.com/EarlBugsBunny/TTTS_Publication). All other data supporting the conclusions of this study are available upon reasonable request.
