# Supplementary material for: Proteomic comparison of intact and fetoscopy-induced fetal membrane defect sites
Source: Pediatr Res. 2025 Dec 26;100(2):863–71. doi: 10.1038/s41390-025-04692-9 (PMC13638478; doi:10.1038/s41390-025-04692-9)
Supplement: Supplementary file 1 [file 41390_2025_4692_MOESM1_ESM.pdf]

Sample Overview

			Donor 1	Donor 2	Donor 3	Donor 4	Donor 5	Donor 6	Donor 7	Donor 8	Total
**Proteomics**	Chorion	Defect				*					**6**
		Control 1				*					**7**
		Control 2				*					**6**
		Control 3				*					**5**
	Amnion	Defect				*					**6**
		Control 1				*					**7**
		Control 2				*					**6**
		Control 3				*					**7**
**SHG**	Collagen										**7**
**Histology**	H&E										**8**
	CD45										**7**
	PKP1										**1**
**Telomer**	Amnion	Defect									**5**
		Control 3									**6**
	Chorion	Defect									**6**
		Control 3									**6**

* Only Full Membrane

Supplemental Table S1: Overview of Samples Used in This Study.

Green indicates samples that were used under the specified condition, while red indicates samples that were excluded. The red marked samples were excluded for technical reasons.

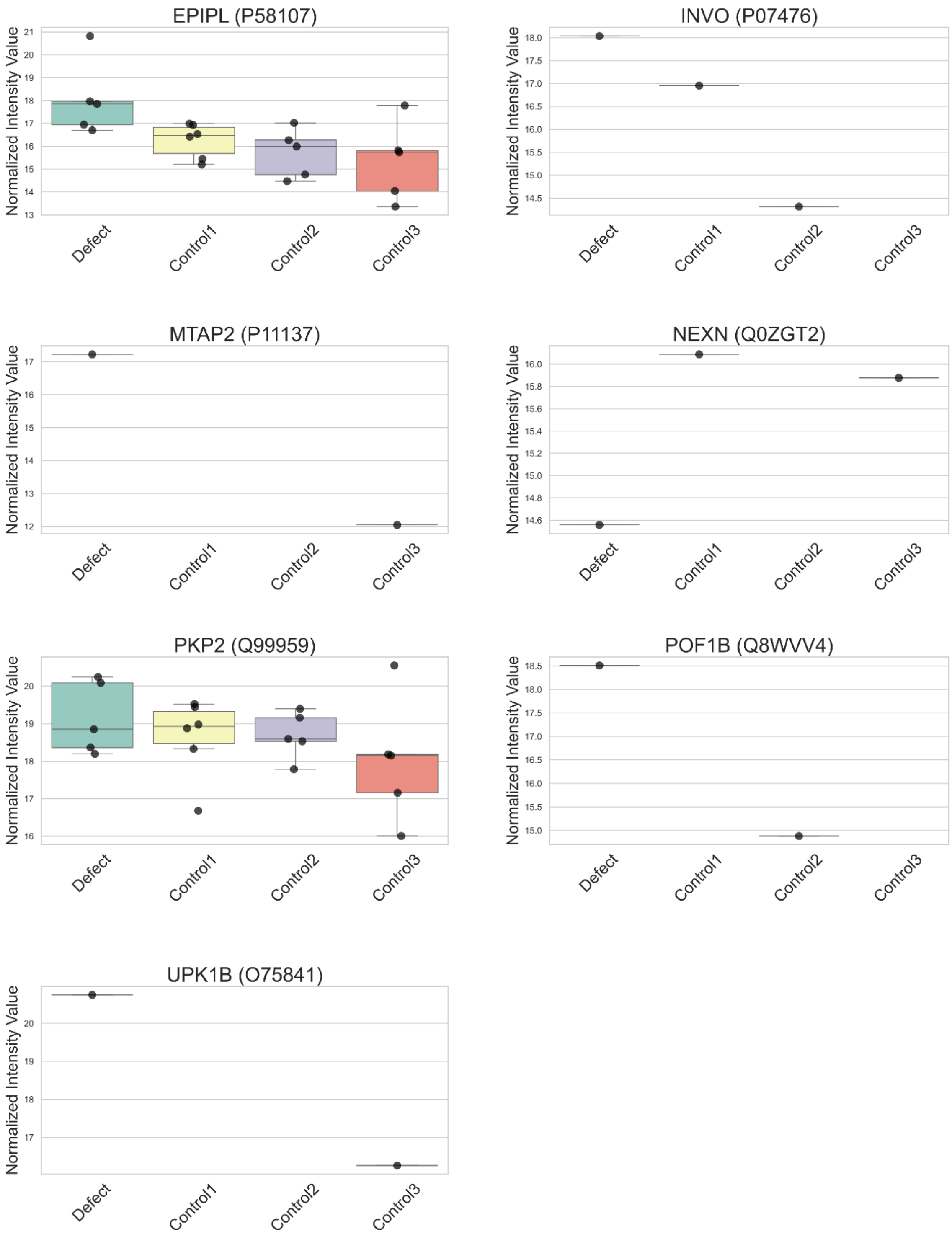

Supplemental Figure S2: Normalized expression of significantly upregulated proteins in defect sites and control sites of the chorion.

Boxplots showing significantly upregulated proteins in defect sites and control sites of the chorion with median and standard deviation. Single points depict the individual donors. If the protein was not detected in a donor, the donor is not represented as a dot in the boxplot

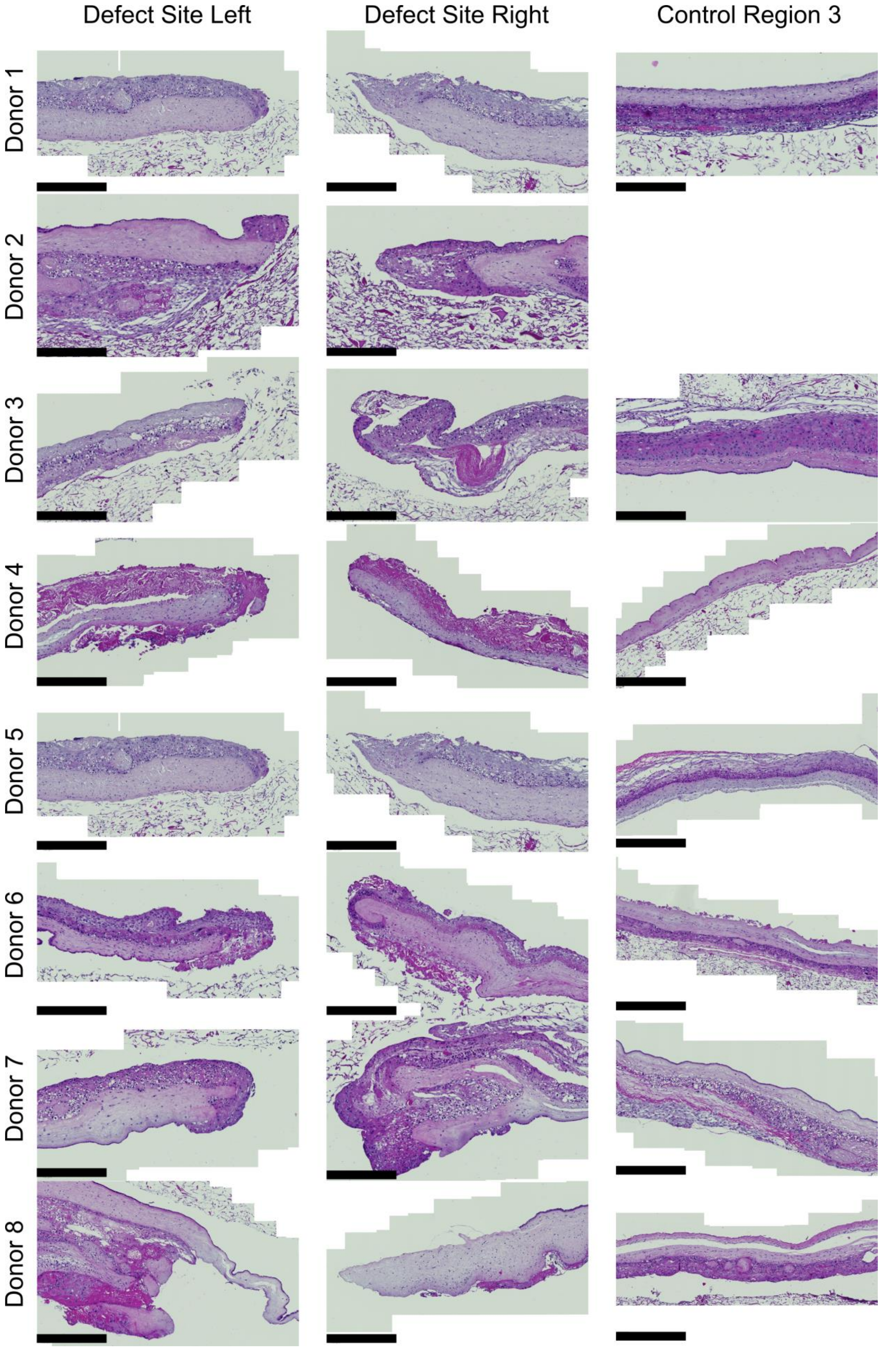

Defect Site Left
Defect Site Right
Control Region 3
Donor 1
Donor 2
Donor 3
Donor 4
Donor 5
Donor 6
Donor 7
Donor 8

Supplemental Figure S3: Hematoxylin and eosin staining of FMs from eight donors.

Each row represents hematoxylin and eosin (H&E) stained FM sections of one donor. Three tissue regions per donor are shown: the left column displays the left defect site of the histology slide, the middle column shows the right defect site, and the right column depicts control region 3. (scale bar = 400 μm)

Donor		1	2	3	4	5	6	7	8
**Intervention [week + day]**		26+1	16+0	16+4	26+4	25+1	19+3	17+0	14+4
**Birth [week + day]**		30+2	32+6	34+2	31+4	32+0	29+3	34+5	33+0
**Induction of Lung Maturation**	Performed?	YES	YES	YES	YES	YES	YES	YES	YES
	Timepoint [week+ day]	25+2	24+4	30+3	26+3*	25+1	25+4	27+5	25+0
**Birth Weight**	Donor [g]	1200	1330	1730	1450	1360	1000	2300	1730
	Acceptor [g]	170	1660	1860	1480	1690	1070	2390	1740
**Birth Outcome**	Donor	live birth	live birth	live birth	live birth	live birth	live birth	live birth	live birth
	Acceptor	intrauterine death	live birth	live birth	live birth	live birth	live birth	live birth	live birth
**Apgar Score**	Donor	1-5-9	7-8-7	3-4-8	8-7-8	4-8-9	3-9-8	8-7-9	2-5-6
	Acceptor	x	4-7-7	5-5-7	8-5-9	4-8-9	3-9-8	8-7-9	2-9-9-

Supplemental Table S4: Neonatal Outcome of the different Donors

Overview of donor and acceptor perinatal outcomes following intervention. All eight cases underwent intervention between 14+4 and 26+4 weeks and delivered between 29+3 and 34+5 weeks. Antenatal lung maturation was performed in all cases (case 4 received a rescue dose shortly before birth, *). All donors were live births; one acceptor (case 1) had intrauterine death. Donor and acceptor birth weights ranged from 1000–2300 g and 170–2390 g, respectively. Apgar scores are shown for each newborn at 1, 5, and 9 minutes.
